# Modeling the Impact of COVID-19 on Dental Insurance Coverage and Utilization

**DOI:** 10.1177/0022034520954126

**Published:** 2020-08-28

**Authors:** S.E. Choi, L. Simon, C.A. Riedy, J.R. Barrow

**Affiliations:** 1 Department of Oral Health Policy and Epidemiology, Harvard School of Dental Medicine, Boston, MA, USA; 2 Office of Global and Community Health, Harvard School of Dental Medicine, Boston, MA, USA; 3 Harvard Medical School, Boston, MA, USA

**Keywords:** simulation model, oral health, health services research, access to care, Medicaid, inequality

## Abstract

Unemployment rates in the United States are rapidly increasing as a result of the COVID-19 pandemic and attendant economic disruption. As employees lose their jobs, many will lose their employer-sponsored dental insurance (ESDI). Changes in insurance coverage are directly related to the oral health of the population, with many at risk of losing access to dental care. We assessed the impact of recent unemployment rates on insurance coverage and dental utilization. We estimated changes in dental insurance coverage at the state level, using previously applied econometric estimates. Expected changes in types of dental procedures performed at dental practices nationwide were assessed using a microsimulation model, using national practice survey data. Changes in emergency department (ED) visits for dental problems were estimated by fitting trendlines to ED visit patterns by payer type. Sensitivity analyses were conducted to assess how variations in unemployment rates and rates of ESDI in response to unemployment could alter the results. Since March 2020, the national unemployment rate has increased by 8.40 percentage points, an increase expected to result in more than 16 million individuals losing ESDI in the United States. Of these individuals, 45.0% are likely to enroll in their state’s Medicaid and Children’s Health Insurance Program, and 47.0% are expected to become uninsured. With these expected changes in dental insurance coverage, the average dental practice would experience decreases in routine checkup visits but increases in tooth extraction, a procedure that is highly used by publicly insured or uninsured patients. In addition, dental-related ED visits would be expected to grow by 4.0%. Losses of employment caused by the COVID-19 in the United States can have countervailing effects on people’s health by impeding access to dental care. Lack of dental insurance is expected to be more pronounced in states that have not expanded Medicaid or do not provide Medicaid dental benefits for adults.

## Introduction

More than 20.5 million jobs have been lost over the past 2 mo as an immediate result of the coronavirus disease 2019 (COVID-19) pandemic, raising the US unemployment rate to 14.7% as of April 2020, the highest level since the Great Depression (US Bureau of Labor Statistics 2020a). One estimate has suggested that the unemployment rate could reach as high as 30% during the second quarter of the year (Soergel 2020). Along with the numerous uncertainties and financial losses associated with loss of employment, many of the unemployed will also lose their employer-sponsored dental insurance (ESDI) in the United States.

While national rates of dental insurance remain lower than those of medical insurance, more than 26 million Americans have some form of dental coverage, with half receiving that coverage through their employer (National Association of Dental Plans 2019). Individuals who have lost their ESDI as a result of COVID-19 have several options for sustaining access to dental care. They may elect to retain their previously held employer-sponsored plan through the Consolidated Omnibus Budget Reconciliation Act (COBRA) that often results in large increases in deductible costs (Driscoll and Bernstein 2012). Individuals could elect to purchase an individual plan on the insurance exchanges created by the Affordable Care Act (ACA), although only 12 states and the District of Columbia have opened their individual exchanges for a special open enrollment period. The remaining states rely on a federally administered exchange that has not opened enrollment, barring individual purchase of plans in these states (Schwab 2020).

Some individuals may newly qualify for Medicaid as a result of loss of income, and states have braced for a swell in Medicaid enrollment (Rudowitz 2020). As lower-paying sectors are disproportionately represented among those with the highest rates of unemployment during the pandemic, some newly unemployed individuals may already have been Medicaid beneficiaries (Burns 2020). Eligibility for Medicaid is highly dependent on whether or not an individual’s state of residence chose to expand Medicaid under the ACA; for unemployed people in the 14 states that have not expanded Medicaid, access to insurance is much less likely (Gangopadhyaya and Garrett 2020).

These changes in insurance coverage will have an impact on dental service utilization and thus on oral health outcomes. Medicaid coverage for dental procedures varies considerably at the state level, and insurance plans offered on exchanges are not required to include dental coverage (Nasseh et al. 2013). Dental insurance is protective against poor oral health outcomes, and individuals without dental insurance are less likely to receive preventive dental services (Wehby et al. 2019). Being uninsured and being a Medicaid beneficiary have both been associated with increased rates of emergency department (ED) utilization for dental problems due to lack of access to usual dental care (Kelekar and Naavaal 2019).

In this study, we estimated the impact on dental insurance coverage in the US population as a consequence of a drastic increase in unemployment rates and thus loss of ESDI during the COVID-19 outbreak. As a result of changes in insurance status, we modeled expected changes in the types of dental procedures performed at US dental practices and in the number of dental-related ED visits based on usual dental utilization patterns by payer type.

## Methods

### Study Design and Model Parameters

We estimated change in dental insurance coverage based on recently increased unemployment and changes in types of dental procedures and dental-related ED visits due to the estimated changes in dental insurance coverage (Appendix Fig. 1). With the rapidly evolving situation around the COVID-19 pandemic, a simulation model provides a framework to estimate future outcomes accounting for uncertainty around model parameters and to predict the results of hypothetical scenarios. We first estimated changes in ESDI rates due to recent increases in unemployment claims, using previously published econometric estimates (Garrett and Gangopadhyaya 2020). These estimates used in our base-case scenario were obtained from a study fitting individual-level regression models on the American Community Survey (ACS) (US Census Bureau 2018) (Appendix Table 1). Given the estimated changes in the ESDI rates due to unemployment, the number of adults and children likely to obtain marketplace coverage, enroll in Medicaid, or become uninsured was estimated using the distribution of dental insurance coverage types by state without ESDI and applying these ratios to the estimated number of losing ESDI in each state.

With the expected changes in insurance coverage, we estimated changes in dental procedures at a general dental practice, using a previously developed microsimulation model, which uses nationally representative data on dental utilization patterns by insurance type (Choi et al. 2020). We estimated changes in the number of dental-related ED visits due to the change in insurance coverage by obtaining the rates of dental-related ED visits among private, Medicaid, and uninsured populations based on a prior analysis of the Nationwide Emergency Department Sample (NEDS) (American Dental Association 2019). From 3-y estimates (2014 to 2016) after the ACA, we fitted trendlines to the distribution for dental-related ED visits by payer and used predicted estimates for 2019 (Appendix Text 1) to incorporate varying rates of ED visits by insurance type. To reflect the current decline in ED visits after the COVID-19 based on the Centers for Disease Prevention and Control (CDC) report (Hartnett et al. 2020), we used the most recent number of overall ED visits (26% below the corresponding week in 2019) as our base-case value.

For modeling types of dental procedures, we simulated a representative sample of 10,000 general dental practices with a number of simulated patient visits assigned. Then for each visit, an insurance type and indicator variables for receiving certain types of procedures were assigned, matching the overall distribution of procedure utilization rates by insurance type. For simulation, we used Monte Carlo sampling with replacement from probability distributions around the patient volume (Robert and Casella 2009). This process accounted for the correlation among utilization rates by insurance type to capture the common co-occurrence of procedures. Analysis was performed in R (v.3.3.2; The R Foundation for Statistical Computing).

### Model Assumption

When estimating changes in dental insurance coverage due to the increased unemployment rates in the United States, we assumed the response of employer-sponsored insurance rates to the unemployment rate would be the same between medical and dental insurance plans. Changes in types of procedure and dental-related ED visits were modeled based on estimated changes in insurance coverage and typical utilization patterns by type of insurance coverage. Throughout our analysis, we assumed that adults age 65 or older who are beneficiaries of a Medicare Advantage plan would not experience changes in insurance coverage due to loss of employment.

### Data Sources

Data sources and input data for the model are detailed in Appendix Table 2. We obtained the state-level dental insurance coverage information from National Association of Dental Plans (2019) and up-to-date number of unemployment claims since March 14, 2020, from the US Bureau of Labor Statistics (Chiwaya and Wu 2020; US Bureau of Labor Statistics 2020b). For dental practice characteristics, annual patient volume was obtained from the American Dental Association (2016, 2017). Utilization rates by procedure and by insurance type were based on Medical Expenditure Panel Survey (MEPS) (Appendix Table 3 and Appendix Fig. 2) (Agency for Healthcare Research and Quality 2018). Because MEPS does not provide procedure-level utilization rates, we grouped Code on Dental Procedures and Nomenclature (CDT) codes into the procedure categories used in MEPS for better interpretation (Appendix Table 4).

### Outcome Metrics

The primary outcome was changes in dental insurance coverage from increase in unemployment rates due to COVID-19. Our secondary outcome metrics included changes in types of dental procedures expected in general dental practices and percentage change in ED visits for dental problems.

### Sensitivity Analyses

The base-case scenario is estimated based on up-to-date increases in unemployment claims information, which varies from 3.9 to 14.9 percentage points across the states. To account for rapidly changing US unemployment rates, we varied the increase in unemployment rates from 5 to 30 percentage points for all states to estimate expected changes in insurance coverage under varying unemployment rates.

Second, while our base-case scenario provides the most conservative response in employer-sponsored insurance rates to unemployment rates, we evaluated the impact of 2 additional response rates to changes in unemployment (Chiwaya and Wu 2020): a time-series model using data from 1998 to 2018 in the National Health Interview Survey (NHIS) (Chiwaya and Wu 2020; Rae et al. 2020) and state-year-level regression models reported in a prior study using Current Population Survey data from 1990 to 2003 (Holahan and Garrett 2009) (Appendix Table 1).

Third, because patients may experience more advanced oral diseases due to a lack of access to dental care at the height of COVID-19, we evaluated scenarios in which patients received more interventive procedures by increasing utilization of nonpreventive procedures (procedures excluding routine oral exam, cleaning, X-rays, fluoride application) by 5% to 20%. Moreover, because individuals who recently had lost ESDI may have different access behaviors than individuals who had been covered by Medicaid or uninsured, we simulated individuals who recently lost ESDI maintaining 50% of access behaviors (50% of the difference in utilization between ESDI and other insurance groups) rather than following access behaviors in Medicaid or uninsured groups.

Last, as emergency care has recently seen a rebound in patient visits since the nadir after the COVID-19 outbreak, we estimated changes in the number of dental-related ED visits with an increase in patient volume by varying the rebound percentage from 80% to 110% of pre-COVID-19 patient volume.

## Results

### Base-Case Analyses

**Figure 1.**
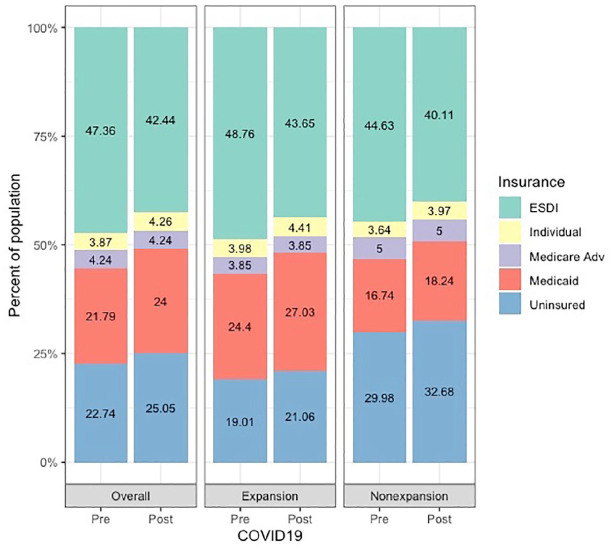
Estimated distributions of dental insurance coverage before and after the COVID-19. “Individual” insurance includes marketplace. ESDI, employer-sponsored dental insurance; Medicare Adv, Medicare Advantage.

As of May 7, 2020, on average, the nationwide unemployment rate increased by 8.40 percentage points. With this increase in unemployment rate, more than 16 million individuals were estimated to lose ESDI (4.91 percentage point decrease from pre-COVID-19 levels) (Table 1 and Fig. 1). Of these individuals, 45.02% would be expected to newly enroll in Medicaid or the Children’s Health Insurance Program (CHIP), 7.98% would purchase individual coverage through an exchange, and the rest (46.98%) are expected to become uninsured, resulting in 24.00% and 25.05% of population enrolling in Medicaid or becoming uninsured, respectively (Fig. 1). Changes in insurance coverage varied between states that expanded under the ACA compared to nonexpansion states. In expansion states, among individuals who are expected to lose ESDI, 51.49% are expected to enroll in Medicaid, with 40.11% becoming uninsured. In nonexpansion states, 59.53% would become uninsured and 33.23% would be expected to enroll in Medicaid.

**Table 1.** Estimated Changes in Dental Insurance Coverage for Overall Population.

State	Increase in Unemployment, Percentage Points	Change in ESDI, Count (Percentage Points)	Change in Marketplace, Count (Percentage Points)	Change in Medicaid, Count (Percentage Points)	Change in Uninsured, Count (Percentage Points)	Change in ED Visits, %
Base case (unemployment as of May 7, 2020)						
Overall population	8.40	–16,127,913 (–4.91)	1,288,131 (0.39)	7,261,351 (2.21)	7,578,431 (2.31)	4.01
Expansion states	8.74	–11,063,724 (–5.11)	929,381 (0.43)	5,696,630 (2.63)	4,437,713 (2.05)	4.41
Nonexpansion states	7.75	–5,054,747 (–4.52)	365,635 (0.33)	1,679,978 (1.50)	3,009,134 (2.69)	3.32
Sensitivity analyses						
Increase in unemployment rate (percentage points)						
5	5.00	–9,597,724 (–2.92)	766,567 (0.23)	4,321,231 (1.31)	4,509,926 (1.37)	2.39
10	10.00	–19,195,447 (–5.85)	1,533,134 (0.47)	8,642,462 (2.63)	9,019,851 (2.74)	4.78
15	15.00	–28,793,171 (–8.77)	2,299,701 (0.70)	12,963,694 (3.95)	13,529,777 (4.12)	7.17
20	20.00	–38,390,895 (–11.70)	3,066,268 (0.93)	17,284,925 (5.27)	18,039,703 (5.50)	9.56
25	25.00	–47,988,618 (–14.62)	3,832,834 (1.17)	21,606,156 (6.58)	22,549,628 (6.87)	11.95
30	30.00	–57,586,342 (–17.54)	4,599,401 (1.40)	25,927,387 (7.90)	27,059,554 (8.24)	14.34
Effect of unemployment on employer-sponsored insurance rates						
Time-series regression	8.40	–27,302,726 (–8.32)	2,180,659 (0.66)	12,292,643 (3.74)	12,829,424 (3.91)	6.80
State-year regression	8.40	–25,967,926 (–7.91)	2,074,049 (0.63)	11,691,669 (3.56)	12,202,207 (3.71)	6.46
Rebound in ED patient volume						
Rebound to 80%	8.40	–16,127,913 (–4.91)	1,288,131 (0.39)	7,261,351 (2.21)	7,578,431 (2.31)	4.23
Rebound to 90%	8.40	–16,127,913 (–4.91)	1,288,131 (0.39)	7,261,351 (2.21)	7,578,431 (2.31)	4.75
Rebound to 100%	8.40	–16,127,913 (–4.91)	1,288,131 (0.39)	7,261,351 (2.21)	7,578,431 (2.31)	5.28
Rebound to 110%	8.40	–16,127,913 (–4.91)	1,288,131 (0.39)	7,261,351 (2.21)	7,578,431 (2.31)	5.81

ED, emergency department; ESDI, employer-sponsored dental insurance.

Table 2 provides estimated changes in insurance coverages due to unemployment rates at the state level. Georgia was the state with the highest increase in unemployment rate by 14.92 percentage points, and our model predicted that 58.87% of those who lost their ESDI would become uninsured, a 5.12 percentage point increase from the pre-COVID-19 level. Even in a state with the lowest increase in unemployment rate, Utah, the uninsured population would be expected to increase by 1.72 percentage points.

**Table 2.** Change in Dental Insurance Coverages by State.

State	Increase in Unemployment, Percentage Points	Change in ESDI Count (Percentage Points)	Change in Marketplace Count (Percentage Points)	Change in Medicaid Count (Percentage Points)	Change in Uninsured Count (Percentage Points)
All	8.40	–16,127,913 (–4.91)	1,288,131 (0.39)	7,261,351 (2.21)	7,578,431 (2.31)
Expansion states					
Alaska	9.88	–42,079 (–5.75)	2,485 (0.34)	21,208 (2.90)	18,385 (2.51)
Arizona	6.70	–284,414 (–3.91)	18,978 (0.26)	124,50 (1.71)	140,927 (1.94)
Arkansas	6.00	–105,513 (–3.50)	6,257 (0.21)	52,134 (1.73)	47,122 (1.56)
California	9.63	–2,224,800 (–5.63)	180,405 (0.46)	1,367,620 (3.46)	676,775 (1.71)
Colorado	6.03	–203,136 (–3.53)	17,047 (0.30)	94,791 (1.65)	91,298 (1.59)
Connecticut	7.65	–159,753 (–4.48)	16,148 (0.45)	90,177 (2.53)	53,428 (1.50)
Delaware^a^	7.68	–43,823 (–4.50)	3,877 (0.40)	10,181 (1.05)	29,765 (3.06)
District of Columbia	9.47	–39,396 (–5.58)	5,957 (0.84)	33,439 (4.74)	0 (0.00)
Hawaii	14.24	–117,721 (–8.31)	22,179 (1.57)	95,542 (6.75)	0 (0.00)
Idaho	6.23	–64,757 (–3.62)	3,827 (0.21)	17,793 (1.00)	43,137 (2.41)
Illinois	6.15	–455,226 (–3.59)	38,758 (0.31)	215,365 (1.70)	201,103 (1.59)
Indiana	8.20	–321,959 (–4.78)	34,101 (0.51)	161,783 (2.40)	126,075 (1.87)
Iowa	7.70	–141,589 (–4.49)	10,711 (0.34)	60,505 (1.92)	70,374 (2.23)
Kentucky	13.93	–363,901 (–8.15)	24,896 (0.56)	190,698 (4.27)	148,307 (3.32)
Louisiana	10.49	–284,431 (–6.12)	14,286 (0.31)	149,313 (3.21)	120,832 (2.60)
Maine	7.72	–61,060 (–4.54)	4,447 (0.33)	23,893 (1.78)	32,721 (2.43)
Maryland^a^	6.77	–239,781 (–3.97)	26,138 (0.43)	122,474 (2.03)	91,169 (1.51)
Massachusetts	9.18	–371,687 (–5.39)	38,656 (0.56)	200,271 (2.91)	132,761 (1.93)
Michigan	12.04	–703,373 (–7.04)	72,791 (0.73)	390,833 (3.91)	239,749 (2.40)
Minnesota	8.57	–282,051 (–5.00)	29,357 (0.52)	129,145 (2.29)	123,549 (2.19)
Montana	7.37	–46,066 (–4.31)	1,706 (0.16)	17,582 (1.65)	26,778 (2.51)
Nebraska	4.91	–55,337 (–2.86)	8,290 (0.43)	20,109 (1.04)	26,938 (1.39)
Nevada	10.47	–188,680 (–6.13)	12,895 (0.42)	72,640 (2.36)	103,145 (3.35)
New Hampshire	10.52	–84,072 (–6.18)	9,631 (0.71)	27,427 (2.02)	47,014 (3.46)
New Jersey	9.71	–504,188 (–5.68)	47,862 (0.54)	217,813 (2.45)	238,514 (2.69)
New Mexico	5.51	–67,463 (–3.22)	3,663 (0.17)	41,055 (1.96)	22,745 (1.08)
New York	8.87	–1,012,229 (–5.20)	61,929 (0.32)	634,443 (3.26)	315,857 (1.62)
North Dakota	7.28	–32,441 (–4.26)	1,291 (0.17)	5,928 (0.78)	25,221 (3.31)
Ohio	7.91	–540,514 (–4.62)	49,024 (0.42)	269,973 (2.31)	221,517 (1.90)
Oregon	7.03	–173,647 (–4.12)	10,988 (0.26)	72,726 (1.72)	89,934 (2.13)
Pennsylvania	10.44	–782,658 (–6.11)	79,245 (0.62)	434,558 (3.39)	268,855 (2.10)
Rhode Island	11.22	–69,915 (–6.60)	7,852 (0.74)	50,640 (4.78)	11,423 (1.08)
Utah	3.93	–72,922 (–2.27)	5,301 (0.17)	12,369 (0.39)	55,252 (1.72)
Vermont	9.01	–33,093 (–5.30)	2,387 (0.38)	16,181 (2.59)	14,525 (2.33)
Virginia	6.80	–339,152 (–3.97)	38,737 (0.45)	101,187 (1.19)	199,229 (2.33)
Washington	10.91	–486,543 (–6.39)	46,279 (0.61)	246,076 (3.23)	194,188 (2.55)
West Virginia	6.33	–66,597 (–3.72)	3,356 (0.19)	34,090 (1.90)	29,151 (1.63)
Nonexpansion states					
Alabama^a^	8.87	–253,891 (–5.18)	8,740 (0.18)	50,149 (1.02)	195,003 (3.98)
Florida	7.88	–992,382 (–4.62)	80,647 (0.38)	427,227 (1.99)	484,508 (2.26)
Georgia	14.92	–923,463 (–8.70)	69,589 (0.66)	310,245 (2.92)	543,629 (5.12)
Kansas	7.10	–120,418 (–4.13)	8,583 (0.29)	30,616 (1.05)	81,220 (2.79)
Mississippi	7.23	–125,294 (–4.21)	5,489 (0.18)	40,208 (1.35)	79,597 (2.67)
Missouri	7.53	–269,866 (–4.40)	21,531 (0.35)	80,241 (1.31)	168,094 (2.74)
North Carolina	7.05	–431,879 (–4.12)	28,021 (0.27)	156,477 (1.49)	247,381 (2.36)
Oklahoma	8.34	–192,205 (–4.86)	11,189 (0.28)	65,677 (1.66)	115,340 (2.91)
South Carolina	8.35	–251,313 (–4.88)	17,472 (0.34)	95,238 (1.85)	138,603 (2.69)
South Dakota	4.06	–20,888 (–2.36)	1,492 (0.17)	5,330 (0.60)	14,066 (1.59)
Tennessee^a^	6.21	–247,529 (–3.62)	22,257 (0.33)	71,866 (1.05)	153,406 (2.25)
Texas	5.68	–959,087 (–3.31)	68,977 (0.24)	278,369 (0.96)	611,741 (2.11)
Wisconsin	7.38	–251,251 (–4.32)	27,405 (0.47)	106,639 (1.83)	117,207 (2.01)
Wyoming	4.95	–16,721 (–2.89)	1,049 (0.18)	2,899 (0.50)	12,772 (2.21)

ESDI, employer-sponsored dental insurance.

a States do not provide dental benefits under Medicaid. Individuals enrolling into Medicaid in these states would be uninsured for any dental care.

**Figure 2.**
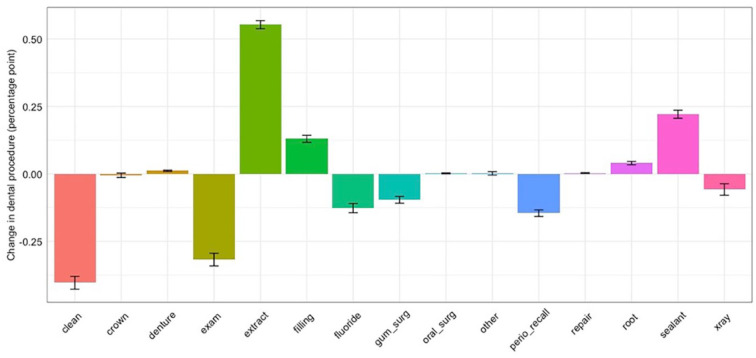
Estimated change in types of dental procedures. exam: diagnostic; clean: prophylaxis; xray: radiographic image; root: root canal; gumsurg: periodontal scaling, root planning, or gum; extract: extraction/tooth pulled; repair: repair of bridges/dentures or relining.

Based on estimated changes in insurance coverage, the number of dental-related ED visits would be expected to increase by 4.01% due to increases in Medicaid enrolled and uninsured populations (Table 1). This increase in dental-related ED visits would be more pronounced in expansion states. For types of procedures performed at dental practices, a substantial decrease would be expected in utilization of diagnostic examination and preventive care, such as cleanings and fluoride application, due to a decline in privately insured patients who visit dental practices for routine checkups and cleanings at a higher rate than publicly insured or uninsured patients (Fig. 2). However, the rate of tooth extraction is expected to substantially increase by 0.55 percentage points (95% confidence interval [CI]: 0.54, 0.56), followed by sealants and restorations, types of procedures more frequently used by publicly insured and uninsured patients, potentially owing to less frequent routine dental visits.

### Sensitivity Analyses

When the overall increase in unemployment rates was varied from 5 to 30 percentage points, the estimated number of individuals losing ESDI ranged from 9.58 to 57.6 million people (Table 1). If the overall unemployment rate is expected to increase by 10 percentage points (a small increase from the current unemployment rate of an 8.4 percentage point increase from the pre-COVID-19 period), more than a half of the US population would be expected to be covered by public insurance or uninsured. The number of dental-related ED visits would be expected to increase by 2.39% to 14.34% within the evaluated unemployment rate increases.

When we assessed the impact of using higher response rates to the unemployment rates obtained from time-series regression and state-year regression models, the expected number of individuals losing ESDI based on the current unemployment rates was between 25.9 and 27.3 million people as compared to 16.1 million in the base case. The number of dental-related ED visits was also estimated to increase at higher rates, by 6.46% and 6.80%, using the time-series and state-year regression models, respectively.

If patients experienced increases in utilization of nonpreventive procedures from 5% to 20% due to the closure of dental clinics, the rate of tooth extraction is expected to increase by 1.14 (95% CI: 1.13, 1.15) to 2.93 (95% CI: 2.93, 2.94) percentage points, compared to 0.55 percentage points (95% CI: 0.54, 0.56) in the base case (Appendix Fig. 3). Moreover, when individuals who recently lost ESDI maintained 50% of access behaviors, patterns of changes in the types of procedures performed remained the same with decreased diagnostic and preventive procedures and increased tooth extraction and sealants but to a lesser degree (Appendix Fig. 4).

When emergency care experienced rebound in patient visits from 80% to 110% of pre-COVID-19 patient volume, the number of dental-related ED visits would be expected to increase by 4.23% to 5.81%.

## Discussion

Our study estimated the impact of rising unemployment rates due to COVID-19 on dental insurance coverage and found that among individuals who lost ESDI due to the current increase in the unemployment rate, 45.02% of individuals are likely to enroll in state-based Medicaid and CHIP programs, and 46.98% are expected to become uninsured, with higher rates in states that have not expanded Medicaid. As a result of these expected changes in dental insurance coverage, dental practices would experience decreases in visits for routine care such as periodic dental exams but increases in procedures such as tooth extraction that are more highly used by publicly insured or uninsured patients than those with private dental insurance. In addition, ED visits for dental problems would be expected to grow.

Given the unprecedented scale of job loss during the COVID-19 pandemic, Medicaid, particularly in expansion states, will play a critical role in supporting access to health care for unemployed workers and low-income families. The steep increases in Medicaid coverage expected as a result of unemployment will likely strain state budgets, restricting already limited resources in communities hardest hit by the pandemic. As occurred during the 2007 to 2009 recession, states may turn to cutting Medicaid dental benefits for adults to reduce strain on growing Medicaid budgets. An additional consideration is reducing reimbursement rates under Medicaid, a major factor in low rates of dentist participation in Medicaid programs (McKernan et al. 2015). Any such changes would likely have even more dramatic impacts on rates of dental access and ED utilization by further restricting the affordability of dental care for low-income patients.

Guidance issued by the CDC in March 2020 reiterated recommendations previously supplied by the ADA to shift to providing only emergency care (American Dental Association 2020a; CDC 2020). While practices in some states in various stages of reopening have begun offering more routine dental care, overall rates of dental production remain much lower than prior to the pandemic, and the prolonged reduction in income has affected the viability of many practice settings (American Dental Association 2020b). As practices reopen, our model anticipates higher rates of more invasive procedures that are more likely to generate aerosols, such as extractions and restorations requiring use of a high-speed handpiece. Personal protective equipment (PPE) shortages may affect providers’ ability to deliver these needed services, especially as infection control recommendations to prevent COVID-19 transmission evolve and add to the expense of operating a dental practice.

From a patient perspective, dental care presents the highest level of financial barriers compared to other health care services, particularly for people without insurance, those with high deductibles, and those in worse conditions requiring procedures covered at a high copayment (Vujicic et al. 2016). With a likely impending recession with COVID-19, economic inequality today will only prolong and worsen considering the disproportionate burden of the pandemic on low-skilled workers (Initiative on Global Market 2020). And without coverage of cost sharing (copayment, coinsurance, or deductible), receiving dental care will be a greater burden for the population affected by COVID-19, exacerbating already profound oral health disparities in the United States. For Medicaid beneficiaries, only 35 states currently provide at least limited dental benefits under Medicaid, with the rest of the states providing either emergency only or no dental benefits (MADPAC 2015). In states where dental benefits are not secured under Medicaid, access to dental providers and services for low-income people would be even more challenging due to COVID-19.

Our analysis has several limitations. First, we assumed the response of employer-sponsored insurance rates to the unemployment rate would be the same between medical and dental insurance plans when estimating changes in dental insurance coverage. However, due to overall lower dental insurance enrollment rates compared to medical insurance, the response rate of ESDI rates to unemployment rates is likely to be higher than that of medical insurance. Thus, our estimates provide conservative results. Second, the projected changes in types of procedure and dental-related ED visits were based on estimated changes in insurance coverage and utilization patterns by type of insurance coverage, which were modeled based on typical utilization patterns prior to COVID-19 due to lack of real-time data on utilization patterns after the COVID-19. This assumption undermines the changes in patient behavior seeking for dental care due to the current circumstance that our study provides unidimensional aspects of changes in access to dental care and utilization as a result of increased unemployment rates due to the COVID-19 pandemic in the United States, and it does not capture the full spectrum of expected changes. Third, because we did not incorporate estimated changes in dental utilization by specialists and assumed that adults age 65 y or older who are beneficiaries of a Medicare Advantage plan would not experience changes in insurance coverage due to loss of employment, the impact of COVID-19 on dental services received by the patients would likely to be greater than what our study projected; thus, our study provides conservative results. Furthermore, we lacked sufficiently rigorous data to expand our model to incorporate regional variation in service utilization and payer mix, such as urban versus rural or by state. Dentist supply, dental care demand, and payer distributions vary a lot across geographic location.

## Conclusion

In summary, our analysis found that the rapid rise in unemployment related to the COVID-19 pandemic will result in substantial loss of ESDI. Roughly half of these individuals will become uninsured; those who become eligible for Medicaid may have variable dental coverage based on the state in which they live. Such shifts may have an impact on dental service utilization and on dental-related ED visits. These changes may further affect dental practice as the dental industry resumes care delivery in uncertain economic and health care environment.

## Author Contributions

S.E. Choi, contributed to conception, design, data acquisition, analysis, and interpretation, drafted and critically revised the manuscript; L. Simon, contributed to conception, design, data acquisition, and interpretation, drafted and critically revised the manuscript; C.A. Riedy, contributed to conception, design, and data interpretation, critically revised the manuscript; J.R. Barrow, contributed to conception, design, data acquisition, and interpretation, critically revised the manuscript. All authors gave final approval and agree to be accountable for all aspects of the work.

## Supplemental Material

DS_10.1177_0022034520954126 – Supplemental material for Modeling the Impact of COVID-19 on Dental Insurance Coverage and Utilization Click here for additional data file. Supplemental material, DS_10.1177_0022034520954126 for Modeling the Impact of COVID-19 on Dental Insurance Coverage and Utilization by S.E. Choi, L. Simon, C.A. Riedy and J.R. Barrow in Journal of Dental Research
